# Origin and Expansion of the Serine Protease Repertoire in the Myelomonocyte Lineage

**DOI:** 10.3390/ijms22041658

**Published:** 2021-02-07

**Authors:** Stefanie A. I. Weiss, Salome R. T. Rehm, Natascha C. Perera, Martin L. Biniossek, Oliver Schilling, Dieter E. Jenne

**Affiliations:** 1 Comprehensive Pneumology Center (CPC-M), Institute of Lung Biology and Disease (iLBD) Helmholtz Zentrum München and University Hospital of the Ludwig-Maximilians University (LMU), 81377 Munich, Germany; stefanie.ai.weiss@gmail.com (S.A.I.W.); salome.rehm@yahoo.de (S.R.T.R.); 2 ARTTIC Innovation GmbH, 80333 Munich, Germany; perera@arttic-innovation.de; 3 Institute of Molecular Medicine and Cell Research, Faculty of Medicine, University of Freiburg, 79104 Freiburg, Germany; martin.biniossek@mol-med.uni-freiburg.de; 4 Institute of Surgical Pathology, University Medical Center Freiburg, Faculty of Medicine, University of Freiburg, 79106 Freiburg, Germany; oliver.schilling@mol-med.uni-freiburg.de; 5 German Cancer Consortium (DKTK), German Cancer Research Center (DKFZ), 69120 Heidelberg, Germany; 6 Max Planck Institute of Neurobiology, 82152 Planegg-Martinsried, Germany

**Keywords:** serine proteases, trypsin ancestor, complement factor D, proteinase 3, cleavage specificity

## Abstract

The deepest evolutionary branches of the trypsin/chymotrypsin family of serine proteases are represented by the digestive enzymes of the gastrointestinal tract and the multi-domain proteases of the blood coagulation and complement system. Similar to the very old digestive system, highly diverse cleavage specificities emerged in various cell lineages of the immune defense system during vertebrate evolution. The four neutrophil serine proteases (NSPs) expressed in the myelomonocyte lineage, neutrophil elastase, proteinase 3, cathepsin G, and neutrophil serine protease 4, collectively display a broad repertoire of (S1) specificities. The origin of NSPs can be traced back to a circulating liver-derived trypsin-like protease, the complement factor D ancestor, whose activity is tightly controlled by substrate-induced activation and TNFα-induced locally upregulated protein secretion. However, the present-day descendants are produced and converted to mature enzymes in precursor cells of the bone marrow and are safely sequestered in granules of circulating neutrophils. The potential site and duration of action of these cell-associated serine proteases are tightly controlled by the recruitment and activation of neutrophils, by stimulus-dependent regulated secretion of the granules, and by various soluble inhibitors in plasma, interstitial fluids, and in the inflammatory exudate. An extraordinary dynamic range and acceleration of immediate defense responses have been achieved by exploiting the high structural plasticity of the trypsin fold.

## 1. Introduction: Distinction between an Ancient Trypsin and Chymotrypsin Cluster

Amide bonds in proteins are very slowly hydrolyzed by surrounding water molecules under natural conditions. However, this slow spontaneous hydrolysis reaction can be accelerated by proteinaceous catalysts, known as proteases, which improve the interaction of a specifically bound water molecule with an amide bond. In contrast to many other posttranslational modifications such as protein phosphorylation, methylation, lipidation, or oxidation, proteolysis cannot be proficiently reversed by religation of the respective peptide fragments and is irreversible. Proteases are common and widespread enzymes and have been classified according to their catalytic mechanism of action as serine, cysteine, metallo- and aspartyl-proteases. Serine-type proteases fall into three categories based on their completely unrelated fold but share in each case the same geometry of the catalytic serine and histidine residues [1].

**Figure 1:**
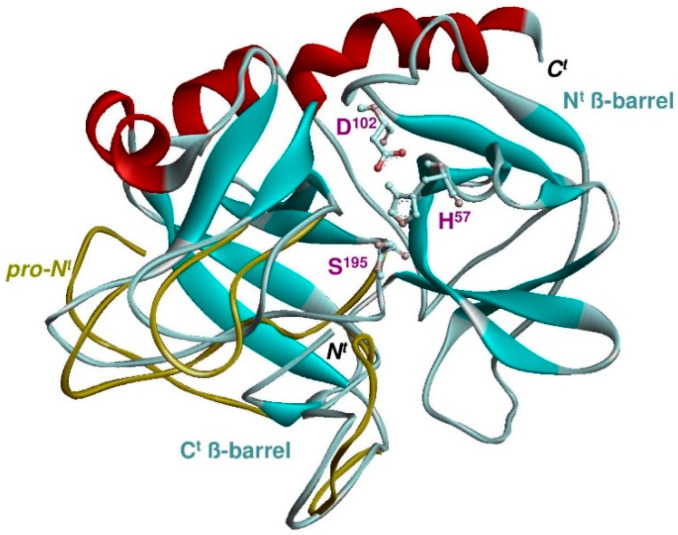
Cα-main chain structure of a serine protease compared with its zymogen (light blue and yellow loops) showing the N- and C-terminal ß-barrels (cyan) with helices in red. The residues of the catalytic triad (His, Asp, and Ser) are shown as ball and stick models and map to the N^t^ (His, Asp) and C^t^ (Ser) ß-strand barrel. After removal of the N-terminal propeptide, the free N-terminus (N) of the mature enzyme (Ile16 to Gly19) inserts into a pocket of the zymogen and forms a salt bridge with Asp194 (chymotrypsinogen numbering). Extensive reorientation of three surface loops occurs in the so-called activation domain (yellow loops) within the C-terminal ß-barrel (C^t^). The S1 pocket opens completely and can now bind the P1 side chain of the substrate. The specificity of the S1 pocket is determined by a few residues of the enzyme directly in contact with the side chain (adapted from [3]).

In chymotrypsin/trypsin-like proteases, a third conserved aspartate residue, Asp102 (chymotrypsinogen numbering), complements the His57 and Ser195 residues and stabilizes the catalytic center of the enzyme (Figure 1). The amino acid residues of chymotrypsin/trypsin-like serine proteases are organized as two six-stranded ß-barrels which form two weakly homologous subdomains with only a few short helical segments on the surface [2]. The His57 and Asp102 residues are located in the N-terminal and the Ser195 in the C-terminal ß-barrel. A linker region (residues 115 to 132 and the C-terminal amphipathic, mainly positively charged helix, connects the N- and C-terminal ß-barrels. The side chain of the substrate residue in the P1 position, next to the scissile peptide bond, interacts with the wall and the bottom of the S1 binding pocket. Further enzyme-substrate hydrogen bonds at positions P1 and P3 and with the Gly193 residue of the enzyme help to position the scissile peptide bond for the attack by Ser195. 

The carbonyl carbon of the amide bond between the P1 and P1′residues is attacked by the polarized hydroxyl group of the Ser195. The N-terminal fragment of the substrate forms a transient covalent complex, while the C-terminal fragment of the substrate is released. A water molecule occupies a position just vacated by the N-terminal P1′residue of the leaving fragment. The latter hydrolyses the acyl-enzyme complex and releases the N-terminal fragment with P1 at its C-terminus. In canonical inhibitor complexes, the reactive site peptide bond remains intact or is slowly cleaved. By contrast, suicide inhibitors, i.e., serpins, form a distorted stable acyl-enzyme complex, in which water molecules cannot attack the acyl bond. In effect, many factors, preferential binding to an optimal substrate, rapid generation of the tetrahedral intermediate, the acylation, and the de-acylation reaction of the entire cycle determine the catalytic efficiency of the enzyme [1]. 

It is generally believed that the ancestors for these different serine protease lineages were more promiscuous than the more specialized present-day members and encompassed the entire repertoire before their specialization. However, by reviewing the literature on the evolution of regulatory serine proteases in the immune system for this Special Issue, we observed an opposite trend and came to a different view. The ancestor of small serine proteases inside our body was highly specific and tightly controlled by multiple interactions with transiently formed substrate complexes. Novel duplicates from this ancestor acquired new functions and specific interactions with just a few mutations, as exemplified with changes of the S1 substrate-binding pocket. The entire repertoire of target functions and serine protease specificities was broadened by the appearance of duplicated copies over evolutionary times. Additional layers of adaptive regulation were introduced by exploiting the structural versatility of a highly specialized trypsin precursor circulating in the blood plasma. As important posttranslational modifiers of various endogenous host proteins, simple serine proteases without additional non-catalytic domains regulate and determine numerous biological processes, including embryonic development and differentiation, cell proliferation, apoptosis, tissue remodeling, wound healing, cell migration, angiogenesis, and immune defense reactions.

**Figure 2:**
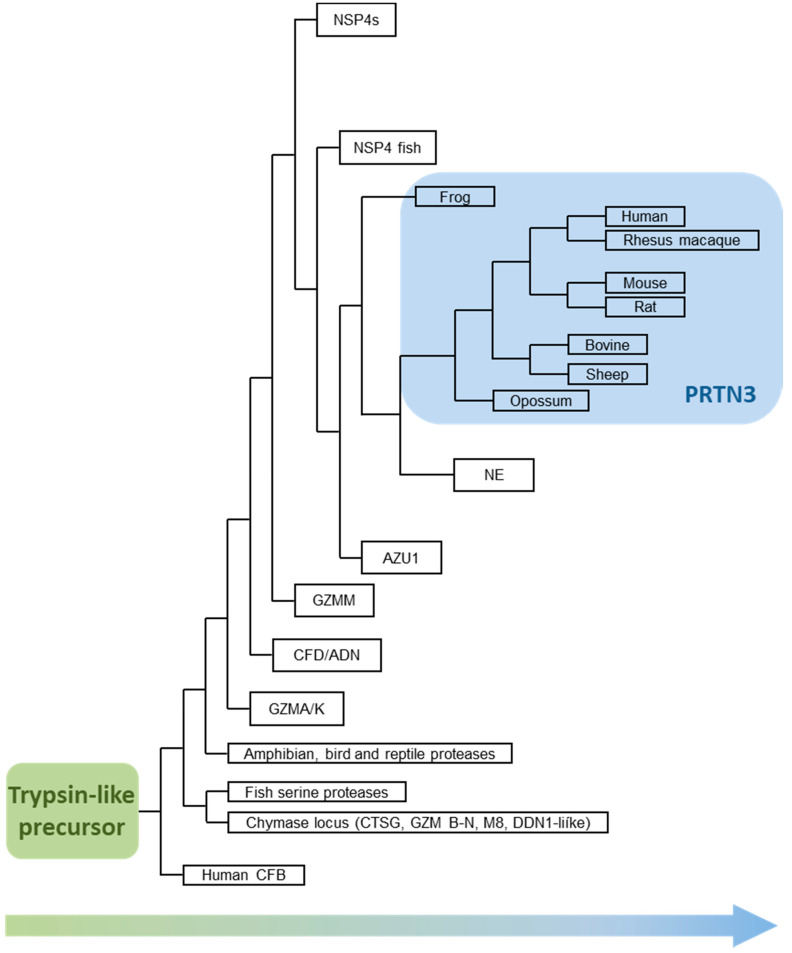
Phylogenetic tree of serine protease evolution towards PRTN3. (adapted from [10]).

The trypsin/chymotrypsin family of serine proteases, the so-called S1A clan (EC 3.4.21), is a very old and diverse group of amide bond hydrolyzing enzymes [4] and can be found in all animal kingdoms (Figure 2) [5,6]. The human genome contains about 100 genes with a catalytically active trypsin-like domain. Most members of the S1A family only consist of a single catalytic domain and are, therefore, regarded as simple serine proteases. Complex type serine proteases are extended by unrelated additional domains primarily at the N-terminus and interact with several other proteins in complex patterns. Typical representatives are clotting factors and components of the complement cascade. Complex-type serine proteases, as well as the simple serine proteases of the digestive system, are phylogenetically very old members and met the primary need of early vertebrates, food supply, prevention of blood loss, and humoral defense responses against infectious agents.

The coagulation and complement cascades became progressively more complex with the emergence of jawless and bony fishes and terrestrial animals [7,8]. As the amino acid sequence identities between distant members of the S1A family are similarly low and are not reliable to reconstruct the deep branches of a phylogenetic tree, the exon-intron pattern and the phase of the introns inserted into the mature coding sequence has been used to define these deep phylogenetically old S1A branches and to assign new S1A members to them [9].

While simple serine proteases of the digestive system in the gastrointestinal tract represent two distinct old branches of the S1A tree, the chymotrypsin and the trypsin cluster [4], other simple serine proteases of the cellular defense system, of the skin and the reproductive system with specificity shifts towards chymotrypsin and elastase-like properties are actually derived from a genuine trypsin ancestor. The earliest divergence of S1A serine proteases was triggered by processes essential to survival and life, exploiting and digesting organic material in extracellular compartments. The chymotrypsin and trypsin clusters of the digestive system are the deepest evolutionary branches of the S1A family. The conversion of trypsin to a similarly active artificial chymotrypsin turned out to be a major challenge for bioengineers [11] and required multiple structural modifications of the S1A fold even in regions that do not directly contact the substrate [12]. These structural differences between the present-day chymotrypsin and trypsin members occurred during a very long evolutionary process and were guided by the strong need for a very efficient catalyst irrespective of other flanking residues around the cleaved amide bond. By contrast, the evolutionary derivatives from the ancestral trypsin were tailored and selected for more specific tasks in body regions that only tolerate a certain degree of limited proteolysis.

## 2. Simple Serine Proteases of Vertebrates are Trypsin Descendants

Complex type serine proteases of coagulation and clotting system and the simple digestive proteases are synthesized by epithelial cells of the liver, called hepatocytes [13], and specialized glandular cells of the pancreas [14]. Hepatocytes are grouped together to form functional liver lobules and are able to target newly synthesized proteins to the basolateral compartments from where they finally reach the venous portal blood flow. All types of S1A serine proteases are synthesized as inactive precursors (zymogens), some are destined for constitutive secretion, e.g., at the basolateral membrane of the hepatocyte, and some are stored as zymogens in intracellular granules of epithelial cells. In response to specific stimuli, these zymogens are locally and transiently converted to their active forms by limited proteolysis on demand [15]. Premature or inappropriate activation of zymogens can be disastrous when the blood supply to vital organs like the heart or the brain is blocked by clotting. Intracellular activation of trypsin can, for example, result in cell death and can cause relapsing-remitting pancreatitis, as seen in familial cases associated with mutations of the trypsin gene [16,17].

Vertebrate trypsinogens, therefore, possess a highly conserved unusual propeptide motif to prevent auto-activation by small amounts of trypsin in the wrong place and at the wrong time during biosynthesis and storage. This tetra-aspartate-lysine motif (DDDK-) is not determined by the substrate specificity of the membrane-bound enteropeptidase, the physiological activator of trypsinogen in the duodenum. The well-conserved tetrapeptide motif in cationic trypsinogens primarily functions as a non-target of trypsin-like enzymes, optimally suppressing self-activation and activation by trypsin-like enzymes [18]. Hence another serine protease, enteropeptidase or also called enterokinase, not affected by acidic residues in the S4 to S2 positions, is required to fulfill this function on the epithelial surface of the duodenum. Fortunately, this highly conserved sequence helps identify a cationic trypsinogen gene in urochordata (protochordata) species [19], in *Ciona intestinalis* and *Ciona savignyi*, which are tunicates and the closest relatives of vertebrates (see Ghost database, Ciona intestinalis genomic and cDNA resources. Available online: http://ghost.zool.kyoto-u.ac.jp/cgi-bin/gb2/gbrowse/kh/ accessed on 08 February 2021). Ciona species not only possess a typical trypsin sequence with a three aspartate-lysine propeptide motif but also carry a putative homolog (Genbank AK116731 partial sequence) encoding the transmembrane enteropeptidase for its activation.

**Figure 3:**
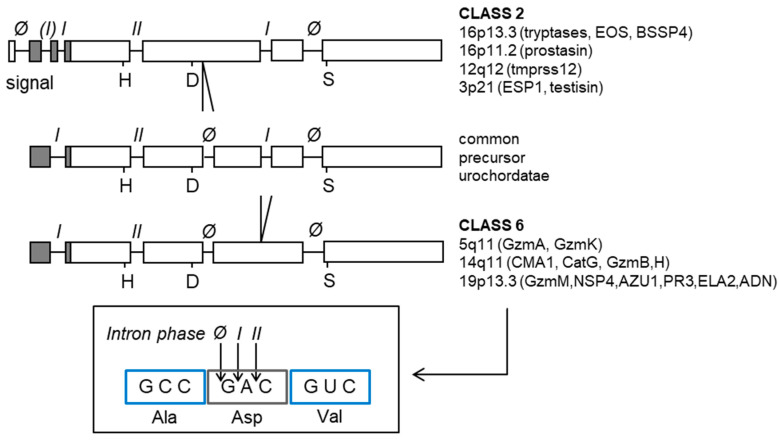
Exon-intron structure of different classes of proteases. Class 2 serine proteases (upper row) lost an intron of phase Ø (zero) close to Asp102 compared to the common precursor protease of urochordatae (middle row). Class 6 serine proteases lost an intron of phase I at position 149 compared to the common precursor (bottom row).

The exon-intron structure of the ciona cationic trypsin gene is similar to the present day bovine cationic trypsin homolog, except for the presence of an additional intron inserted between residue 109 and 110 (chymotrypsinogen numbering) just seven residues downstream of the catalytically important Asp102. This intron position is shared with all members of granule-associated serine proteases of hematopoietic cell lineages, lymphocytes, mast cells, and granulocytes [20]. Another feature shared with these latter, simple serine proteases, is the absence of a fifth disulfide bond bridging the Cys128 within the barrel linker with the Cys232 of the C-terminal helix. Comparing the exon-intron structure of the ciona trypsin gene with that of glandular kallikreins (KLK1-KLK15), trypsins (PRSS1 to PRSS4), mast cell tryptases and testisin, it becomes quite clear that most if not all simple serine proteases except for those of the very old digestive system are descendants of an ancient trypsin precursor (Figure 3). Hence it is not surprising that the vast majority of these simple catalysts prefer basic residues at the P1 position like their ancestor. While class 2 S1A members have lost the phase-zero intron close to the Asp102, class 6 members retained this ancestral intron and are missing the phase-1 intron at residue position 149 (chymotrypsin numbering). 

The critical evolutionary change took place when serine proteases were utilized and acted in the intravascular, peri- and intercellular space of various tissues and organs. Tight control of catalytic activities was mandatory and had to be restricted by fine-tuned space and time limits. Cellular toxicity had to be contained by intracytoplasmic inhibitors. Toxicity during biosynthesis and transport out of the ER had to be prevented by propeptide sequences that were not susceptible to endogenous enzymes before the zymogens reached a safe subcellular compartment or the bloodstream and a remote site for specific local activation.

## 3. Repurposing of the Early Trypsin Ancestor, Complement Factor D

The primitive digestive gland in arthropods and mollusks is a single organ, called the hepatopancreas [21], which fulfills the functions of both the liver and pancreas in mammals. During mammalian embryogenesis [22] the pancreas and liver develop from diverticula of the foregut in the region of the later duodenum and develop into separate organs with both endocrine and exocrine functions. At an early stage of vertebrate evolution, the pancreas already specialized in the production and delivery of digestive enzymes to the intestinal tract, the duodenum, in particular trypsins, while the liver parenchyma functioned as an endocrine gland synthesizing and delivering various highly abundant proteins directly to the bloodstream. Among the plasma proteins are transport proteins, lipoproteins, coagulation and complement factors, and a simple trypsin-like protease called complement factor D (CFD).

CFD, also known as adipsin (ADN) [23], is constitutively produced by hepatocytes and adipocytes and occurs at 50 µg/mL concentrations in human plasma [24], according to other sources, its concentration is 1.8 µg/mL, the lowest of any complement protein [24]. In contrast to the trypsin zymogen, it is secreted as a mature and fully processed serine protease, which has some esterolytic, but lacks proteolytic (amidolytic) activities [25]. Its S1 substrate-binding pocket is shared with classical trypsin-like enzymes, most of which show an acidic Asp189 residue at the bottom of the S1 pocket. The positively charged arginine side chain at position P1 of the substrate is attracted by the negatively charged Asp189. This interaction, as well as five enzyme-substrate hydrogen bonds at positions P1 and P3 and Gly193, help to position the scissile peptide bond for the nucleophilic attack by the polarized hydroxyl group of Ser195. Sequence recognition around the cleaved peptide bond is often not very restricted and depends on additional interactions between the protease, the substrate, and substrate-bound cofactors.

As a fully mature protease, CFD can be freely distributed in the circulation and other body fluids without risk and negative consequences. It is not encountered by the many highly abundant serine protease inhibitors of blood plasma, like serpins and α2-macroglobulin. The circulating levels of CFD are too low to directly activate the alternative pathway via its genuine substrate target. The reason for this unusual property of the mature molecule is the inactive self-inhibited active site conformation. This catalytically silent conformation is transformed into the active conformation by binding to its cognate substrate, the nascent surface-bound C3bB complex [26]. CFD only interacts efficiently with the open conformation of complement factor B (CFB) and activates the latter in the C3bB complex, thereby enhancing the cleavage of C3 by the C3bBb complex and the covalent deposition of C3b on the surface of foreign or altered host target cell membranes [26]. In this way, CFD enhances pathogen and immune complex recognition [27] and elimination as well as acute and chronic inflammation [28,29,30].

Although CFD evolved very early in vertebrate evolution and is found in the genomes of bony fishes and all land-living animals, complete deficiency of CFD (ADN) does not increase the general susceptibility to microbial infections [31,32,33]. Unexpectedly, the anti-microbial defense system is only marginally affected and cannot cope so efficiently with specific types of Neisseria infections, resulting in meningococci meningitis in affected young children [34] and gonococcal infections in CFD-deficient young women. However, the high degree of consanguinity in these very rare familial cases should be wisely taken as a warning signal, as other homozygous mutations in these individuals may also affect the immune system negatively. Likewise, CFD (ADN)-deficient mice display no apparent abnormality in their development and body weight but a kinetically impaired opsonization capacity of *Streptococci pneumoniae* in vitro [35]. In the event of a meningococcal outbreak in humans, children would have a high mortality rate due to meningitis and sepsis without the action of CFD. In young adults and later in life, cross-reactive antibodies to atypical meningococci of the mouth flora have formed and can substitute for this deficiency.

As CFD was also discovered as a highly expressed gene transcript in adipocytes [36], strongly upregulated locally by tumor necrosis factor-alpha (TNF-α), its contribution to inflammation has recently been studied in a mouse model of inflammatory arthritis, suggesting that the recruitment and organ infiltration of neutrophils is accelerated by CFD and its enhancing effects on the alternative complement pathway [37]. Conversely, higher levels of circulating ADN lower the risk of developing type 2-diabetes, as ADN or products of the alternative complement pathway appear to prevent dedifferentiation and cell death of beta cells in the pancreas [38]. High levels of CFD reduce lethality from specific life-threatening infections but can increase collateral tissue damage in autoimmune conditions. Long-term beneficial effects of ADN on insulin-producing beta cells, on the other hand, decrease the risk of future diabetes. The beneficial action of ADN on beta cell survival appears to depend on continuous local complement turnover and production of C3a, which interacts with C3a-receptor 1 on beta cells of the pancreas. The ancestral ADN, derived from a common trypsin precursor in the pancreas, may have exerted this local complement-mediated positive effect on beta cell function in the pancreas quite early and retained this function throughout evolution. Appropriate acute responses to pathogens and acceptable damage to the host by the associated inflammation is a delicate balance, as pointed out by Medzhitov [39]. Host tolerance to invading pathogens and acceptability of tissue damage by protease-dependent immune responses must be adjusted to the specific pathogen and the way how it attacks the host.

## 4. The Structural Basis for New S1 Specificities

The vast majority of classic type S1A serine proteases, according to the Barrett nomenclature [40], cleave after arginine or lysine residues within a flexible surface loop of a folded protein. The reason for this predominant chemical property is the Asp residue at the bottom of the so-called S1 pocket in most members of the S1A family. The distinction between the two naturally occurring basic P1 residues is not very pronounced. Trypsin-type S1 pockets form a direct ionic interaction with the arginine side chain, while the lysine side chain is connected to the Asp189 in the S1 pocket by a bridging water molecule via hydrogen bonds. Ser190 forms an additional hydrogen bond to both lysine and arginine side chains. The S1-P1 interaction dominates the recognition of the substrate, and other side chains of the substrate recognized by the S2 to S4 pockets of the protease do not matter very much for the selectivity towards basic P1 residues [41]. Systemic dissemination of constitutively active and fully maturated trypsin-like serine proteases would be very dangerous, as they may interfere with complement and coagulation cascades. Hence all complement and coagulation proteins with a trypsin-like domain circulate as inactive zymogens and are activated in a stepwise manner by other factors on demand. In addition, these enzymes, once activated, require cofactors and the appropriate substrate in the correct conformation. The activity of mature CFD is likewise optimally controlled by conformational transitions of both the substrate and the enzyme itself, thereby avoiding unintended proteolysis in the body [26,42].

There are only very few examples in present-day serine proteases, in which the negative charge of the S1 pocket has been shifted from position 189 to 226 (chymotrypsinogen numbering) without a loss of its preference for basic P1 side chains. In the human genome, the only examples are CFD, CFB, and a C1r-like serine protease, called C1r-LP. The aspartate residue at position 189 is replaced by serine residues in factor C2 and C1r-LP and by asparagine in CFB [43,44,45]. The C1r-related serine protease is synthesized as a complex-type zymogen and is able to cleave the haptoglobin precursor chain already very early in the ER after an arginine residue [44,46]. Limited proteolysis during biosynthesis in the ER is potentially cytotoxic and therefore requires a highly restricted and tightly regulated processing enzyme-like C1r-LP. Most processing of secretory proteins takes place at a later stage in the secretory pathway in the trans-Golgi complex or in pre-lysosomal acidic compartments.

Repositioning of the negatively charged aspartate from the bottom to the wall of the S1 pocket has been comprehensively studied in the anionic rat trypsin D189G/G226D mutant [47] and was shown to retain the cleavage specificity of the mutant after basic residues. Relocation of the negative charge to the pocket wall, however, altered the relative preference of the mutant trypsin in favor of lysine over arginine residues at the P1 site. Adjacent residues at positions Ser190 and Ser228 are able to interact with Asp226 and can partially shield its negative charge. This repositioning of negative charge opened the door for more versatile changes of P1 specificities and for the binding of hydrophobic residues during vertebrate evolution. However, the rapid expansion of serine protease specificities necessitated other means to protect cells and tissues against serine proteases during biosynthesis in the expressing host cells and after their secretion into the peri- and extracellular space. Regulation of these new proteolytic activities could not take place in the extracellular environment by integrating these specificities into the existing complicated cascades of cleavages after arginine residues.

## 5. Substrate Profiling of Neutrophil Serine Proteases

The last five decades have brought us an enormous growth of knowledge about the specificity of purified and recombinant proteases in vitro and in vivo. Substrates in the extended conformation form an antiparallel ß-sheet with the peptide backbone of proteases, in particular with the residues Gly193, Ser195, Ser214, and Gly216. This basic interaction with the main chain of the protease is mediated by three and two hydrogen bonds at positions P1 and P3, respectively, which positions the scissile peptide bond for the nucleophilic attack by the polarized hydroxyl group of Ser195. In most instances, target specificities for proteins and oligopeptides differ and depend on the extended peptide sequence and not only on main chain contacts and the interaction with the side chain of the P1 residue. N- and C-terminal to the scissile amide bond peptide, substrates interact with the protease surface at more distant sites, which are also known as extended substrate-binding pockets on the prime (S2 to S5) and nonprime position (S1′ to S2′). The differential binding specificity of the S1 pocket and proteolytic activity can be affected, e.g., by a conformational change of the so-called 99-loop, which is induced by the side chain of the P2 residue of the substrate. Binding specificity and subsite cooperativity has been unraveled by various combinatorial methodologies, which have been reviewed recently [48,49,50].

Specificity profiling by different methods has created a large number of in vitro data, which are of limited use to predict protease functions in vivo. In parallel, quantitative mass spectrometry methods and whole proteome techniques have been advanced and used to understand complex proteolytic events in vivo associated, e.g., with wound healing [51] and inflammatory responses. Identification of cleavage events, fragment sequences, and active proteases is still a major challenge to link potential substrates to the transient activity of a single protease within a network of competing inhibitors, substrates, and functionally similar proteases [50]. Most often, the sequence identified is not only susceptible to a single protease, and the neo-termini of the resulting fragments are modified further in vivo by carboxy- and aminopeptidases. 

Evolutionary innovations with regard to the serine protease repertoire in the myelomonocyte lineage are therefore very difficult to evaluate under in vivo conditions. In addition, the underlying forces, molecular mechanisms, and the selection criteria shaping the fate of duplicated genes at the species and population level are unknown. Although the true ancestral protease for a multigene family cannot be resurrected and directly compared to the present-day proteases, a hypothetical protease precursor for the myelomonocyte proteases can be deduced from the gene repertoire of the extant vertebrates. Functional innovations, context optimization, and dosage adaptations have primarily guided the rapid evolutionary expansion of serine proteases in the myelomonocyte lineage. How nature makes great leaps by very simple steps can be illustrated with the four serine proteases of innate immune cells. In the following, we will focus on the structural changes of the S1 pocket, which led to new protease functions with just a few mutations. The reader more interested in the specificity of these proteases affected by other subsite pockets on the prime side (S1′, S2′) and distant to the S1 site (S4 to S2) is referred to original studies which were performed by the many research groups cited in [51,52,53,54,55,56] and found in our reference list below.

## 6. Conversion of the Trypsin Ancestor into Chymotrypsin/Elastase-Like Enzymes

Diversification of cleavage specificities could not occur with freely circulating complex-type serine proteases of the blood plasma, but in fact, emerged in cell lineages of the immune defense system, in neutrophils, mast cells, lymphocytes, and macrophages. Regulation of limited proteolysis at specific sites of danger and attack by microbes was shifted towards the controlled release of subcellular granules containing these simple but already fully processed active serine proteases. Today, human immune defense cells express a unique set of eleven single domain serine proteases whose zymogens are already constitutively converted to their active form by cathepsin C (CatC, dipeptidyl-peptidase I) during biosynthesis, sorting, and storage in cytoplasmic granules [57,58]. Host cells and tissues are protected from these fully processed serine proteases with broadened specificities by early sequestration and sorting into cytoplasmic granules at low pH and are bound to cationic polymers like heparin sulfate and chondroitin sulfate in an inactive state. Three members expressed by human neutrophils, neutrophil elastase (NE), proteinase 3 (PR3), and cathepsin G (CatG), cleave after small aliphatic and aromatic residues, respectively, a fourth member, neutrophil serine protease 4 (NSP4), which was recently discovered last, cleaves selectively after arginine, but not lysine residues [59,60]. All four members display a negatively charged residue at position 226, but this negative charge is shielded by hydrophobic side chains of residues, primarily valines in position 190 and 216. One member expressed by natural killer cells and cytotoxic lymphocytes, granzyme B (GzmB), moreover, displays an arginine residue at position 226 and thereby an overall positively charged P1 binding pocket. It acquired a caspase-like specificity by convergent evolution and efficiently hydrolyses procaspases after an acidic residue [61].

**Figure 4:**
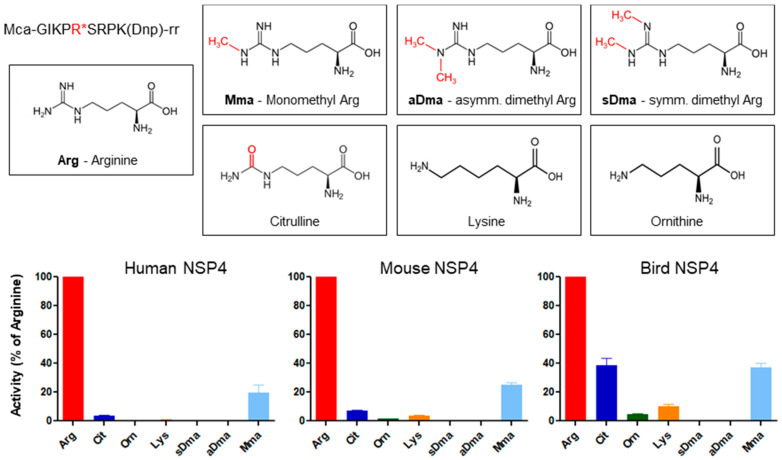
Hydrolysis after unmodified arginine residues is preferred by neutrophil serine protease 4 (NSP4) in birds, mice, and men. The activity of human, mouse, and bird NSP4 was determined by activity assays of 40 nM NSP4 from the different species using specific FRET substrates (λ_ex_ = 325 nm λ_ex_ = 392 nm) (2 µM) as previously reported in [55]. Substrates contained modified arginine residues at position P1. In addition, citrulline (Cit), lysine (Lys), and ornithine (Orn) specific substrates were included in the analysis (Perera N.C., unpublished results).

It is widely believed that present-day proteins acquired their specific activities during recent evolution after gene duplication of promiscuous ancestral precursors, which were multifunctional [62,63]. Likewise, a despecialized universal precursor as the starting point for the evolution of granule-associated serine protease has been postulated [64]. Despecialization is undoubtedly of advantage and a useful functional adaptation in the digestive tract. This phenomenon was reported for a well-characterized serine protease from fiddler crabs, which was given the misleading name collagenase [65]. The trypsin-related collagenase from the hepatopancreas of fiddler crab possesses an aspartate residue at position 226 and can cleave on the carboxyl-terminal side of residues with positively and negatively charged as well as hydrophobic side chains [66]. However, completely novel cleavage specificities can evolve de novo with just a few mutations from an already highly specialized precursor protease such as CFD. Acquiring new target specificities of serine protease during evolution does not appear to be very difficult or rare. Serine proteases can continually gain new specificities and lose their original functions over evolutionary time as long as they are compatible with or even increase the fitness of their host. At the stage of bony fishes, specificities for basic residues still dominated the extracellular tissue and central plasma compartments. However, the specific location and duration of their action were highly restricted. The NSP4, a local duplicate of the CFD gene on human chromosome 19p13.3, was even confined to a strict selectivity for arginine residues [59,67]. The normally deep S1 pocket has structural features of an elastase-like enzyme, as the Asp226 is shielded by Val190 and Val216 and is buried in the S1 pocket. Interaction with an arginine side chain at the P1 position was newly invented by introducing two serine residues at positions 192 and 216 on the surface of the protease, which are conserved among all NSP4 homologs [68,69]. This unprecedented selective recognition of a P1 arginine in a substrate by NSP4 is an extreme form of specialization avoiding adverse cellular effects by cleaving P1 lysine substrates. Cleavage after naturally occurring modified arginine residues, like citrullin, mono-methylated, and di-methylated arginine residues; however, has probably not driven the selection and optimization of these structural features, as the respective substrates with modified arginine residues in P1 are cleaved with much lower efficacy (Figure 4). 

Relocating the Asp residue from position 189 to 226 in the S1 trypsin pocket served as a starting point to introduce a hydrophobic platform for the aliphatic portion of the arginine and for the side chains of other hydrophobic residues. At the stage of bony fishes more than 400 million years ago, immune cell-associated serine proteases with selectivity for basic and hydrophobic side chains already emerged in the lymphocyte and myelo-monocyte lineages [70], which gave birth to the extant Gzm A, K, and M [71] in lymphocytes and NSP4 in neutrophils (Figure 2). The T-cell GZM A/K locus with a conserved Asp189 at the bottom of the S1 together with orthologous flanking genes has been identified in the phylogenetically oldest fish lineage (agnatha) [70], which are jawless and have a cartilaginous skeleton. This locus coding for trypsin-like activities of lymphocytes is well-conserved in all vertebrate lineages, including humans. Replicates of the primordial trypsin locus were most likely generated by two successive whole-genome duplications before the radiation of vertebrate lineage [72]. These replicates were then structurally converted by just a few mutations, which changed the critical determinants of substrate interactions.

## 7. PR3 Preceded Leukocyte Elastase (ELANE)

Phylogenetic analysis of extant granule-associated serine proteases reveals the approximate order of gene duplications within the chromosome 19 locus, showing a common branch for a GzmM/NSP4 precursor and a younger NSP4-derived branch for the NE, PR3, and azurocidin triplet (Figure 2) [10]. Azurocidin was completely lost in the rodent lineage and was converted into an inactive serine protease homolog in other mammalian lineages [73]. Consistent with this phylogenetic tree and its branches, a serine protease gene has been identified in the genome of amphibians, in *Xenopus tropicalis* and *Xenopus laevis*. Its specificity profile resembles most the pattern of human PR3 and, to a lesser extent, the pattern of human NE. The Xenopus precursor prefers Val over Ala in the P1 position and accepts Ile poorly in its S1 pocket, unlike human NE. Its P1 specificity compared to hNE is somewhat more restricted, which may be due to the Ile190, which is larger than the Val190 in hNE. In the egg-laying semiaquatic platypus (*Ornithorhynchus anatinus*), a single PR3/NE-like gene has also been described in addition to genomic loci for GzmM, NSP4, and CFD [74]. Although not yet characterized at the biochemical level, conservation of the typical catalytic triad and, in particular, of the Ile190 suggests, it is functional and resembles hPR3 in its specificity. Duplication of the PR3/NE gene and the emergence of a typical NE have thus occurred just before mammalian radiation.

Larger deviations from the highly restricted NSP4 and CFD activities appear to be associated with the appearance of new serpins capable of controlling elastase-like proteases in response to their release in emergency situations [75]. The evolutionary process has been guided by stepwise permission of novel specificities in conjunction with the appearance or availability of appropriate inhibitors in the cytoplasm of neutrophil precursors and in body fluids outside of these cells [76]. The latest addition to the neutrophil armory of granule-associated serine proteases is NE (ELANE, ELA2), which displays the highest catalytic activity and damaging potential (see Figure 2) [10]. As a mirror image of this trend, NE is most tightly controlled by a very efficient and abundant plasma inhibitor, alpha-1 protease inhibitor (α1PI), which is systemically upregulated several-fold during acute inflammatory reactions [77]. Unsurprisingly, this member of the NSP family attracted the most attention of the pharmaceutical industry as an interesting drug target for half a century, but with very little rewards so far. Topping the high level of elastase control already implemented by Mother Nature turned out to be a real challenge. 

## 8. Functional Divergence of PR3 in Mouse and Man

**Figure 5:**
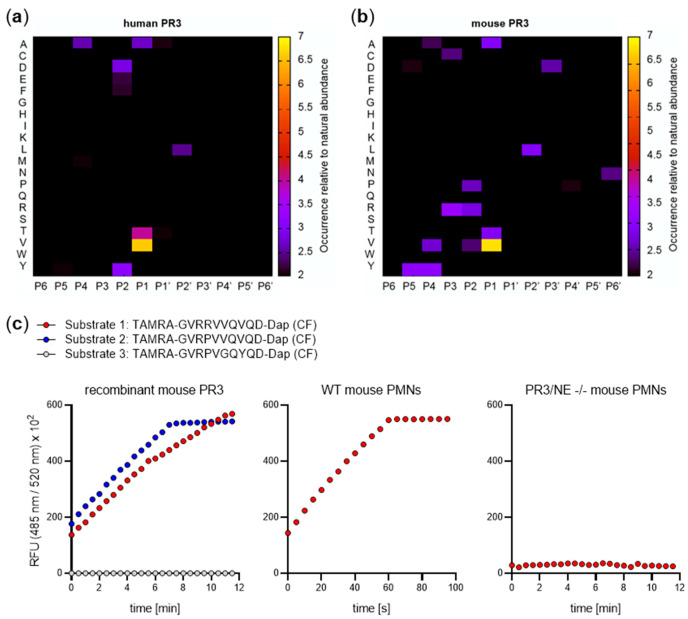
Profiling of the proteinase 3 (PR3) cleavage site reveals the cleavage site specificity of human (**a**) and mouse (**b**) PR3 after V, A, or T at position P1; the peptide libraries were generated with GluC; (**c**) Differences of the mouse PR3 cleavage sites in P1 and P2 position were analyzed by activity assays with 50 nM mPR3 using specific fluorogenic TAMRA substrates (λ_ex_ = 485 nm λ_ex_ = 520 nm) (10 µM) in 50 mM Tris, 150 mM NaCl, 0.01% Tween-20 (pH 7.4).

To investigate PR3-related inflammatory processes and diseases, in vivo studies are indispensable. Although the amino acid sequence homology between mouse and human PR3 is relatively high at 68%, differences in the critical substrate binding sites determine altered specificity. This has to be considered when investigating the function and role of PR3 in mouse models. While specific substrates have been developed to discriminate between hPR3 and hNE, no specific substrate that gets cleaved exclusively by mouse PR3 is yet available [78,79]. To profile the active site cleavage specificity, we used proteome-derived peptide libraries [80] as a technique that has previously identified subtle subsite differences in the specificity profiling of human trypsin-isoenzymes [81]. The pattern of human PR3 to cleave after V, T, or A in P1 stated in the MEROPS peptidase database could be confirmed by our analysis (Figure 5a,b). Our data furthermore show evident differences of the mouse PR3 cleavage sites at the P1 and P2 positions. (Figure 5c).

Not only differences in specificity but also the presence and localization of PR3 in mouse and human have to be critically considered. For example, membrane-bound PR3 (PR3^mb^) can be found on human neutrophils, and its amount is stable in each individual while mouse neutrophils do not display PR3 on their surface [82]. Interaction of the neutrophil-specific receptor CD177 with PR3^mb^ of resting neutrophils enhances their activation after anti-neutrophil cytoplasmic antibody (ANCA) binding in humans. Due to the missing CD177 binding site on mouse PR3 and the lack of PR3^mb^ on resting neutrophils in mice, no appropriate model for PR3-associated vasculitis is yet available [83]. Attempts to establish a mouse model with transgenic mice expressing human PR3, as well as human CD177, failed to induce the disease in vivo [84].

The critical role of PR3^mb^ in the disease progression of ANCA-associated vasculitis is further strengthened by the fact that PR3^mb^ is not efficiently inhibited by its main endogenous inhibitor α1PI [85], and the extent of superoxide release from PR3^mb^-rich neutrophils triggered by ANCA binding is higher compared with those having low PR3^mb^ expression [86]. 

## 9. Regulation of Proteinase 3 Activity

In a healthy state, the activity of serine proteases is regulated by endogenous inhibitors of the serpin family—mainly by α1PI. After noncovalent docking to the catalytic site of the protease, a covalent acyl-enzyme complex is formed between Ser-195 of PR3 (chymotrypsinogen numbering) and the carboxy group of the Met358 residue of α1PI. The reactive center loop (RCL) is inserted into a ß-sheet. The protease is translocated to the opposite pole of the serpin and distorted to an inactive state. As a result, the protease is trapped as a pretty stable covalent protease-inhibitor complex [87]. This complex is cleared by receptor-mediated endolysosomal uptake [88]. Another member from the serpin family, monocyte neutrophil elastase inhibitor (MNEI), or serpinB1, contains two protease cleavage sites in the RCL with different specificities for either chymotrypsin-like or elastase-like proteases, including PR3 [89]. Besides its capacity of limiting protease-induced inflammatory damage, serpinB1 also protects neutrophils against caspase-3 mediated cell death by interaction with PR3 [90].

Further molecules that are able to regulate PR3 activity are the canonical inhibitor elafin, which is produced locally at sites of infection, as well as the plasma protease inhibitor α-2-macroglobulin that acts systemically on circulating proteases [91,92].

Downregulation or inactivation of inhibitors under pathological conditions and the resulting imbalance between proteases and anti-proteases contribute to tissue damage and inflammatory cascades. This effect can be mainly attributed to increased PR3 activity since binding studies revealed that α1PI inhibits PR3 significantly less than NE and CatG [93,94]. The reduced affinity to PR3 has been observed for other protease inhibitors as well. Local scarcity of endogenous inhibitors in combination with their lower binding affinity to PR3 results in excessive PR3 activity.

Since PR3 is the main antigen for ANCA in the rare autoimmune disease, granulomatosis with polyangiitis (GPA), the effect of antibodies on PR3 activity has also been the subject of investigation. In fact, 79% of patient ANCA directed against PR3 have been shown to modulate PR3 activity, with the proportion of inhibitory ANCA being higher than activity-enhancing antibodies [95]. However, no correlation of activity-modulating ANCA with the disease status of the patients could be observed. Studies with monoclonal antibodies revealed two mechanisms of how antibody binding can interfere with PR3 activity. A direct inhibitory effect is attained by antibody binding close to the substrate-binding pocket or within the active site cleft of PR3, thereby either partially or fully blocking its substrate binding region. Other antibodies were shown to indirectly inhibit the proteolytic activity by inducing a conformational change through an allosteric mechanism [96]. Recently it was also shown that the dynamics of latent PR3 epitopes are affected by distal mutations. Experimental data and molecular dynamics simulations support the activation of certain antibody binding regions on PR3 due to conformational changes resulting in increased flexibility of loop 3B [97]. Based on these findings, it can be assumed that antibody binding and subsequent allosteric modulation of PR3 make certain epitopes accessible to other antibodies that can, in turn, modulate PR3 activity. 

## 10. Impact of Proteinase 3 on Human Disease

The role of PR3 in disease is most strongly associated with the autoimmune vasculitis GPA, formerly Wegener’s granulomatosis, where it was shown to be the target antigen for ANCA [98]. After priming of neutrophils by pro-inflammatory cytokines, PR3 is transported from azurophilic granules to the membrane where it is accessible to ANCA binding. Antibody binding triggers excessive activation of neutrophils resulting in neutrophil extracellular trap (NET) formation and release of reactive oxygen species, proteolytic enzymes, and cytokines [99,100]. Further recruitment of effector cells like macrophages and neutrophils accelerate inflammation. The resulting self-reinforcing process causes necrotizing granulomatous inflammation and small-vessel vasculitis [101].

Besides its role as an autoantigen, PR3 dysfunction plays a critical pathophysiological role in numerous neutrophil-related diseases. Its ability to cleave several groups of molecules, including structural proteins, receptors, and cytokines, makes PR3 a crucial key player affecting inflammatory cascades in the progress of various diseases.

As the most abundant NSP in circulating neutrophils, it is not surprising that PR3 was shown to be involved in the development of chronic obstructive pulmonary disease (COPD), a disease hallmarked by chronic neutrophilic inflammation of the respiratory tract [102]. Apart from the local protease anti-protease imbalance in the lungs, an increased circulating pool of PR3 activity was also reported in patients suffering from COPD and cystic fibrosis (CF) [103,104,105]. This increased activity results in cleavage and activation of several cytokines, including the chemoattractant IL-8 (CXCL8), TNF-α, and interleukin-1-beta (IL-1β), leading to the potentiation of the inflammatory loop associated with COPD [103,106,107]. The recruitment and accumulation of neutrophils due to the activation of cytokines is further accelerated by PR3-dependent inhibition of the anti-inflammatory pathway triggered by progranulin (PGRN) [99,108]. In addition, the granulin peptides resulting from this cleavage increase the production and release of IL-8, thereby accelerating the feedback loop [109]. A direct pathogenic role of PR3 is the degradation of extracellular matrix (ECM) proteins like elastin, collagen, fibronectin, and laminin resulting in emphysema due to tissue injury and destruction [110].

As for all inflammatory diseases, the above-discussed effects on cytokine activation and neutrophil recruitment also have a critical impact on disease progression in CF. This infection-triggered chronic inflammation of the airways is hallmarked by excessive mucus production combined with impaired mucus clearance. The interaction of PR3 with the complement pathway contributes to this detrimental accumulation of mucus in the airways of these patients. PR3 truncates the N-terminus of complement component 5a (C5a) receptor (C5aR). As a result, C5aR lacks the ability to bind C5a, leading to inactivation of the complement signaling pathway and consequently impaired bacterial clearance [111].

Furthermore, the increased risk for lung and liver diseases in patients suffering from the inherited disorder α1PI deficiency is directly associated with PR3 activity due to the lack of its main endogenous inhibitor.

In general, the impact that PR3 activity has on neutrophil infiltration, signaling, and destruction of structural molecules is associated with numerous diseases reaching from COPD and CF over rheumatoid arthritis to non-alcoholic fatty liver disease [112,113].

## 11. Conclusions

The constitutively produced circulating pool of preformed active serine proteases triggers and accelerates defense reactions at local sites if needed. NSPs, however, can also perpetuate and aggravate severe tissue damage and inflammatory pathologies. Although small molecule or peptide-based inhibitors against individual neutrophil serine proteases have been extensively explored in the past 50 years, efforts of the pharmaceutical industry so far have created very little impact in the clinic except for alpha-1-antitrypsin augmentation therapy in emphysema patients with congenital alpha-1-antitrypsin deficiency. Lowering the activities of all four serine proteases of neutrophils should generate much greater beneficial effects in pathological settings. This therapeutic goal is indeed achievable with cathepsin C inhibitors. Small molecule inhibitors were recently shown to effectively prevent the conversion and storage of NSP zymogens in neutrophils. Their therapeutic applications in neutrophil-driven inflammatory diseases, such as bronchiectasis, chronic obstructive pulmonary disease (COPD), cystic fibrosis, idiopathic interstitial pneumonia, acquired respiratory distress syndrome (ARDS) and psoriasis, are currently under investigation.

## Data Availability

The data presented in this study are available on request from the corresponding author. The data are not publicly available since they were not made publicly available elsewhere.

## Abbreviations

ADN	Adipsin
ANCA	Anti-neutrophil cytoplasmic antibody
AZU1	Azurocidin 1
BSSP4	Brain-specific serine protease 4
C1r-LP	C1r-like serine protease
C5a	Complement component 5a
C5aR	Complement component 5a receptor
CatG	Cathepsin G
CF	Cystic fibrosis
CFB	Complement factor B
CFD	Complement factor D
Cit	Citrullin
CMA1	Chymase 1
COPD	Chronic obstructive pulmonary disease
CTSG	Cathepsin G gene
DDN1	Duodenase 1
ECM	Extracellular matrix
ELA2	Neutrophil elastase
ER	Endoplasmatic reticulum
ESP1	Separin
FRET	Förster resonance energy transfer
GPA	Granulomatosis with polyangiitis
Gzm	Granzyme
GZMA/K	Granzyme A/K gene
GZMM	Granzyme M gene
IL-1β	Interleukin-1-beta
MNEI	Monocyte neutrophil elastase inhibitor
mPR3	Membrane-bound proteinase 3
NE	Neutrophil elastase
NET	Neutrophil extracellular trap
NSP4	Neutrophil serine protease 4
Orn	Ornithine
PGRN	Progranulin
PR3	Proteinase 3
PRTN3	Proteinase 3
RCL	Reactive center loop
TAMRA	Carboxytetramethylrhodamine
Tmprss12	Transmembrane protease serine 12
TNF-α	Tumor necrosis factor alpha
α1PI	Alpha-1 protease inhibitor
